# Diagnosing selective mutism: a critical review of measures for clinical practice and research

**DOI:** 10.1007/s00787-021-01907-2

**Published:** 2021-12-01

**Authors:** Chaya Rodrigues Pereira, Judith B. M. Ensink, Max G. Güldner†, Ramón J. L. Lindauer, Maretha V. De Jonge, Elisabeth M. W. J. Utens

**Affiliations:** 1 Levvel, Academic Center for Child and Adolescent Psychiatry, Meibergdreef 5, 1105 AZ Amsterdam, The Netherlands; 2 Amsterdam UMC, Department of Child and Adolescent Psychiatry, Amsterdam Public Health, University of Amsterdam, Amsterdam, The Netherlands; 3 Faculty of Social Sciences, Department of Education and Child Studies, Clinical Neuroscience and Developmental Disorders, University Leiden, Leiden, The Netherlands; 4 Research Institute of Child Development and Education, University of Amsterdam, Amsterdam, The Netherlands; 5 Department of Child and Adolescent Psychiatry/Psychology, Erasmus MC—Sophia Children’s Hospital, Rotterdam, The Netherlands

**Keywords:** Selective mutism, Assessment, Child anxiety, Multi-informant, Diagnosis

## Abstract

Selective mutism (SM) is an anxiety disorder (prevalence 1–2%), characterized by the consistent absence of speaking in specific situations (e.g., in school), while adequately speaking in other situations (e.g., at home). SM can have a debilitating impact on the psychosocial and academic functioning in childhood. The use of psychometrically sound and cross-culturally valid instruments is urgently needed.

The aim of this paper is to identify and review the available assessment instruments for screening or diagnosing the core SM symptomatology. We conducted a systematic search in 6 databases. We identified 1469 studies from the last decade and investigated the measures having been used in a diagnostic assessment of SM. Studies were included if original data on the assessment or treatment of SM were reported. It was found that 38% of published studies on SM reporting original data did not report the use of any standardized or objective measure to investigate the core symptomatology. The results showed that many different questionnaires, interviews and observational instruments were used, many of these only once. The Selective Mutism Questionnaire (SMQ), Anxiety Disorders Interview Schedule (ADIS) and School Speech Questionnaire (SSQ) were used most often. Psychometric data on these instruments are emerging. Beyond these commonly used instruments, more recent developed instruments, such as the Frankfurt Scale of SM (FSSM) and the Teacher Telephone Interview for SM (TTI-SM), are described, as well as several interesting observational measures. The strengths and weaknesses of the instruments are discussed and recommendations are made for their use in clinical practice and research.

## Introduction

Selective mutism (SM) is a psychiatric condition characterized by persistent failure to speak in specific social situations (usually in school) despite speaking adequately in other situations (usually with close family members). The disorder was categorized as an anxiety disorder in the DSM-5 and ICD-11 [1, 2] (see Table 1 for the current diagnostic criteria for selective mutism), based on multiple studies showing an overlap in behavioral characteristics and etiological factors in children with SM and high comorbidity with other anxiety disorders, specifically social anxiety [3, 4].

**Table 1:** Current diagnostic criteria for selective mutism as described in the DSM-5 and ICD-11

DSM-5 Criteria	
Diagnostic criteria	313.23 (F94.0)
A	Consistent failure to speak in specific social situations in which there is an expectation for speaking (e.g., at school) despite speaking in other situations
B	The disturbance interferes with educational or occupational achievement or with social communication
C	The duration of the disturbance is at least 1 month (not limited to the first month of school)
D	The failure to speak is not attributable to a lack of knowledge of, or comfort with, the spoken language required in the social situation
E	The disturbance is not better explained by a communication disorder (e.g., childhood-onset fluency disorder) and does not occur exclusively during the course of autism spectrum disorder, schizophrenia, or another psychotic disorder

ICD-11 Criteria	
6B06 Selective mutism	
**Description** Selective mutism is characterized by consistent selectivity in speaking, such that a child demonstrates adequate language competence in specific social situations, typically at home, but consistently fails to speak in others, typically at school. The disturbance lasts for at least one month, is not limited to the first month of school, and is of sufficient severity to interfere with educational achievement or with social communication. Failure to speak is not due to a lack of knowledge of, or comfort with, the spoken language required in the social situation (e.g., a different language spoken at school than at home) **Exclusions** Schizophrenia (6A20) Transient mutism as part of separation anxiety in young children (6B05) Autism spectrum disorder (6A02)	

American Psychiatric Association, 2013

World Health Organization, 2018

SM is not as rare as once believed, with reported prevalence rates between 0.7 and 2% [5–7]. The broad prevalence range may be attributed to differences in sampling strategies, such as the inclusion of clinical or community samples, sample characteristics such as age range or immigrant status, or to the diagnostic methods used. SM usually has an onset between 2 and 4 years, but often remains unrecognized until the child enters school [8]. If left untreated, SM can take a chronic course and affect social–communicative capacity, mental health and quality of life in adolescence and adulthood [9–13].

Given the interference with social, communicational and academic development and wellbeing, it is important to identify and treat SM timely and accurately. Valid and reliable diagnostic instruments are needed to further advance research into the behavioral characteristics, possible subgroups, treatment efficacy and long-term outcome of individuals with SM. Several diagnostic instruments have been developed over the last two decades that can be used to classify SM and/or to investigate the severity of symptoms in different contexts. Recommendations for a thorough diagnostic assessment have been described [14] and include information from multiple informants, e.g., parental information, teacher information, direct assessment of the child’s behavior and self-reported information from the child if possible. Some children are able to complete self-report measures or communicate about their difficulties via parents, drawings or cards [14, 15]. The diagnostic instruments for the assessment of SM include questionnaires, structured diagnostic interviews and observational measures to be used in clinical settings, the classroom or in daily life situations. These measures were designed for, or used in studies on, screening, diagnostic assessment and classification or the assessment of symptom severity and treatment progress. Obviously, a full diagnostic assessment involves more than examining speaking behavior alone, and includes the medical and developmental history of the child, family history, parenting and family functioning, life events, the examination of other behavioral difficulties or comorbid psychiatric symptoms and direct assessment of cognitive, academic and language skills [14, 15]. In addition to the core characteristics of SM, e.g., the amount of speaking behavior in different situations, a broader assessment is needed to differentiate SM from other disorders, to investigate comorbid disorders or associated developmental difficulties, and to examine familial and contextual factors that may play a role in the development or persistence of the disorder.

In clinical practice, valid and reliable measures are crucial for an accurate diagnosis which is an essential step for providing effective treatment and evaluate treatment progression [14]. In research, the use of standardized measures improves the comparability of studies. However, currently in clinical practice and in research, different instruments are used to classify SM and to investigate symptom severity, or sometimes no specific measures are used that specifically target SM. Therefore, the aim of this systematic review is to describe and identify which assessment tools have been used for the purpose of screening, classification or monitoring treatment outcomes on speaking behavior and SM symptomatology in in the past decade. In this review  we focus on the core criterion of SM, i.e., speaking behavior (criterion A of the DSM-5 classification). In addition, methodological strengths and limitations of the assessment instruments will be reviewed to provide recommendations to the usefulness of the instruments for clinical practice and research.

## Methods

For this review, we selected all articles about SM that reported original data in the last decade.

### Search strategy and selection criteria

An electronic literature search was performed by an information specialist of a University Medical Library. The following databases were searched three times, in January 2019, March 2020 and July 2021: Embase, Medline (Ovid), PsycINFO, Web of Science, Cochrane Central and Google Scholar. Titles and abstracts were searched with a combination of keywords and subcategories, shown in Table 2.

**Table 2:** Keywords in literature search into instruments for SM

Embase
('selective mutism'/de OR (((selectiv* OR electiv* OR voluntar*) NEAR/3 (mutism* OR mute*))):ab,ti) AND (child/exp OR adolescent/exp OR adolescence/exp OR 'child behavior'/de OR 'child parent relation'/de OR pediatrics/exp OR childhood/exp OR 'child nutrition'/de OR 'child welfare'/de OR 'child abuse'/de OR 'child advocacy'/de OR 'child development'/de OR 'child growth'/de OR 'child health'/de OR 'child health care'/exp OR 'child care'/exp OR 'childhood disease'/exp OR 'child death'/de OR 'child psychiatry'/de OR 'child psychology'/de OR 'pediatric ward'/de OR 'pediatric hospital'/de OR 'pediatric anesthesia'/de OR 'pediatric intensive care unit'/de OR (adolescen* OR preadolescen* OR child* OR kid OR kids OR toddler* OR teen* OR boy* OR girl* OR minors OR underag* OR (under NEXT/1 (age* OR aging)) OR juvenil* OR youth* OR kindergar* OR puber* OR pubescen* OR prepubescen* OR prepubert* OR pediatric* OR paediatric* OR school* OR preschool* OR highschool* OR PICU OR PICUs):ab,ti)
Medline (Ovid)
(Mutism/ OR (((selectiv* OR electiv* OR voluntar*) ADJ3 (mutism* OR mute*))).ab,ti.) AND (exp Child/ OR exp Infant/ OR exp Adolescent/ OR exp "Child Behavior"/ OR exp "Parent Child Relations"/ OR exp "Pediatrics"/ OR "Child Nutrition Sciences"/ OR exp "Child Welfare"/ OR "Child Development"/ OR exp "Child Health Services"/ OR exp "Child Care"/ OR "Child Rearing"/ OR exp "Child development Disorders, Pervasive"/ OR "Child Psychiatry"/ OR "Child Psychology"/ OR "Hospitals, Pediatric"/ OR exp "Intensive Care Units, Pediatric"/ OR (adolescen* OR child* OR kid OR kids OR toddler* OR teen* OR boy* OR girl* OR minors OR underag* OR (under ADJ1 (age* OR aging)) OR juvenil* OR youth* OR kindergar* OR puber* OR pubescen* OR prepubescen* OR prepubert* OR pediatric* OR paediatric* OR school* OR preschool* OR highschool* OR PICU OR PICUs).ab,ti.)
PsycINFO
(Elective Mutism/ OR (((selectiv* OR electiv* OR voluntar*) ADJ3 (mutism* OR mute*))).ab,ti.) AND (100.ag. OR 120.ag. OR 140.ag. OR 160.ag. OR 180.ag OR 200.ag. OR (adolescen* OR infan* OR newborn* OR (new ADJ born*) OR baby OR babies OR neonat* OR child* OR kid OR kids OR toddler* OR teen* OR boy* OR girl* OR minors OR underag* OR (under ADJ1 (age* OR aging)) OR juvenil* OR youth* OR kindergar* OR puber* OR pubescen* OR prepubescen* OR prepubert* OR pediatric* OR paediatric* OR school* OR preschool* OR highschool* OR suckling* OR PICU OR NICU OR PICUs OR NICUs).ab,ti.)
Web of Science
TS = (((((selectiv* OR electiv* OR voluntar*) NEAR/2 (mutism* OR mute*)))) AND ((adolescen* OR preadolescen* OR child* OR kid OR kids OR toddler* OR teen* OR boy* OR girl* OR minors OR underag* OR (under NEAR/1 (age* OR aging)) OR juvenil* OR youth* OR kindergar* OR puber* OR pubescen* OR prepubescen* OR prepubert* OR pediatric* OR paediatric* OR school* OR preschool* OR highschool* OR PICU OR PICUs)))
Cochrane Central
((((selectiv* OR electiv* OR voluntar*) NEAR/3 (mutism* OR mute*))):ab,ti) AND ((adolescen* OR preadolescen* OR child* OR kid OR kids OR toddler* OR teen* OR boy* OR girl* OR minors OR underag* OR (under NEXT/1 (age* OR aging)) OR juvenil* OR youth* OR kindergar* OR puber* OR pubescen* OR prepubescen* OR prepubert* OR pediatric* OR paediatric* OR school* OR preschool* OR highschool* OR PICU OR PICUs):ab,ti)
Google Scholar Top 100 relevant references
"selective|elective|voluntary mutism|mute " adolescents|preadolescents|child|children|kid|kids|toddler|teen|boy|girl|boys|girls|minors|juvenile|youth|kindergarten|puberty|prepuberty|pediatrics|pediatric|paediatric|paediatrics|school|preschool|highschool

Studies were included in this review if they (1) were published in English between 2010 and July 2021 and accessible in full-text format, (2) reported original data on the assessment of SM.

**Fig. 1:**
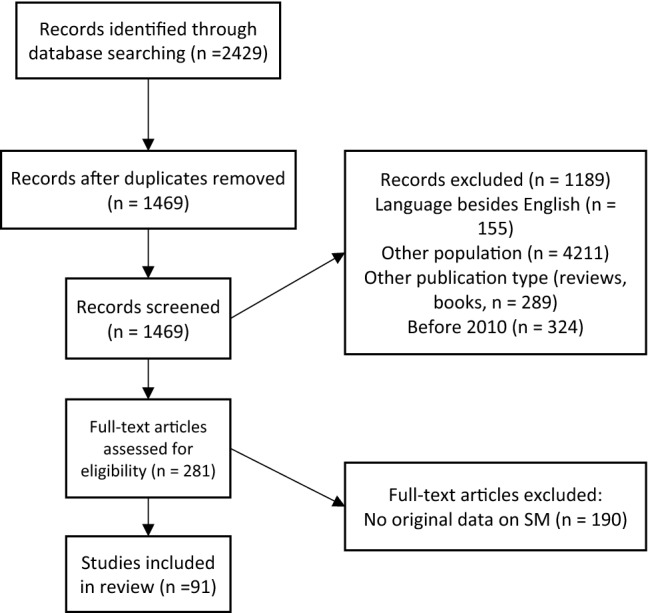
Flowchart

We excluded studies that did not discuss SM, as well as studies that did not report original data on SM, such as reviews and meta-analyses. Doctoral theses, conference papers or abstracts were also excluded. A detailed overview of the study selection process is displayed in Fig. 1. For the psychometric description of the instruments, we searched for source articles describing the instrument and its psychometric properties through reference lists.

In line with the Prisma Guidelines [16], two authors (CRP and JE) independently screened the titles and abstracts of the retrieved citations. A third author (MdJ) was consulted in case the screeners had different ratings or doubt to include or exclude a paper, thereafter consensus was established. In case of doubt on the basis of the abstract screening, the article was included for full-text screening. Next, all selected full-text articles were screened to verify the selection. The data collection from relevant studies was performed using a pre-designed extraction spreadsheet.

## Results

### Search results

Figure 1 shows that (after correction for duplication) 1469 studies were identified. After screening, 1189 studies were excluded because based on the title and abstract the inclusion criteria were not met. Thereafter, the remaining 281 articles were screened in full text and 91 studies were included in the review.

### Descriptive data

From the 91 articles reporting original data on the assessment and/or treatment of children with SM, 35 articles (38%) did not report use of objective or standardized measures for SM symptomatology. In these articles the diagnosis was often based on a clinical assessment. In 56 articles, one or more standardized measures were used to classify SM and/or quantify severity (see Table 3).

**Table 3:** Studies using standardized instruments for assessing SM symptomatology

ID	Author	Year	Sample:	N	Age range	Type of study	Instrument used to assess SM speaking behavior	Format
Treatment studies								
1	Ale et al	2013	SM	2	5	Treatment study	SMQ	Questionnaire
2	Barterian et al	2018	SM	5	5–14	Treatment study	SMQ, DBR	Questionnaire and observation
3	Bergman et al	2013	SM	21	4–8	Treatment study	ADIS, SMQ, SSQ, IEBE, SNAP, CGI	Questionnaires, interview, observation
4	Bork & Bennett	2019	SM	3	8	Treatment study	SMQ, SSQ, DRCB-P/DRCB-T	Questionnaires and observation
5	Bunnell et al	2018	SM	15	5–17	Treatment study	SMQ, ADIS, Shaping hierarchy of speaking behavior observational measure	Questionnaire, interview, observation
6	Bunnell & Beidel	2013	SM	1	17	Treatment study	ADIS, frequency of target behavior	Interview and observation
7	Catchpole et al	2019	SM	31	4–10	Treatment study	ADIS, SM-BOT, SMQ, SSQ, SNAP	Questionnaires, interview, observation
8	Christon et al	2012	SM	1	15	Treatment study	SMQ	Questionnaire
9	Cornacchio et al	2019	SM	29	5–9	Treatment study	ADIS, SMQ, SSQ	Questionnaires and interview
10	Esposito et al	2016	SM	138	6–18	Treatment study	SMQ	Questionnaire
11	Heilman et al	2012	SM/community controls	20/49	3–18	Treatment study	ADIS	Interview
12	Jacob et al	2013	SM	1	4	Treatment study	SMQ	Questionnaire
13	Khan & Renk	2018	SM	1	5	Treatment study	SMQ	Questionnaire
14	Klein et al	2017	SM	40	5–12	Treatment study	ADIS, SMQ, SM-SCCS	Questionnaire, interview, observation
15	Lang et al	2016	SM	36	3–9	Treatment study	SMQ, ADIS	Questionnaire and interview
16	Mitchell & Kratochwill	2013	SM	4	5–10	Treatment study	Severity of Behavior Form, Coded direct observations, GAS	Questionnaire and observation
17	Oerbeck et al	2011	SM	7	3–5	Treatment study	ADIS, SMQ, SSQ,	Questionnaires and interview
18	Oerbeck et al	2014	SM	24	3–9	Treatment study	ADIS, SMQ, SSQ	Questionnaires and interview
19	Oerbeck et al	2015	SM	24*	3–9	Treatment study	ADIS, SMQ, SSQ	Questionnaires and interview
20	Oerbeck et al	2018	SM	30*	8–14		ADIS, SMQ, SSQ, ILC	Questionnaires and interview
21	Ooi et al	2012	SM	5	6–11	Treatment study	SMQ	Questionnaire
22	Plener et al	2012	SM	1	8	Treatment study	ESCM	Questionnaire
23	Reuther et al	2011	SM	1	8	Treatment study	ADIS	Interview
24	Serra et al	2015	SM	1	17	Treatment study	ADIS, SMQ	Questionnaire and interview
25	Skedgell et al	2017	SM	1	6	Treatment study	ADIS, SMQ, SSQ	Questionnaires and interview
Other descriptive studies								
26	Bufferd et al	2010	Community sample	8/541	mean age 3	Community cohort study	PAPA	Interview
27	Carbone et al	2010	SM/anxiety/typically developing comparison groups	44/65/49	mean age 8.2 y; SD = 3.4 y	Case–control observational study	SpSQ-P, SpSQ-T; Verbal and Nonverbal Social Interactions Skills	Questionnaires
28	Cholemkery et al	2014	SM/anxiety/autism spectrum disorder/typically developed	43/140	6–18	Case–control design, validity study	Kinder-DIPS	Interview
29	Diliberto & Kearney	2016	SM	57	Mean = 6.7 y, SD = 1.9	Latent Class	SMQ, ADIS	Questionnaire and interview
30	Diliberto & Kearney	2018	SM (parent-reported)	278*	6–10	Latent Class	SMQ	Questionnaire
31	Edison et al	2011	SM/anxious/non-anxious groups	21/17/25	4–13	Case–control observational study	SpSQ, SM-BCFPI, Observational coding of speech during parent–child interaction laboratory setting	Questionnaire, interview and observation
32	Gensthaler et al	2016	SM/SAD/internalizing/community controls	95/74/46/119	3–18	Cross-sectional	Kinder-DIPS	Interview
33	Henkin et al	2010	SM/community controls	10/10	mean 9.35 (2.59)	Case–control	SMQ	Questionnaire
34	Kamani & Monga	2020	SM/SAD	31	4–14	Retrospective	ADIS, CSR, CGAS, SMQ	Questionnaire, interview and observation
35	Klein et al	2013	SM	33	5–12	Comparative (parent/therapist) one-sample study	Therapist-created questionnaire for DSM-IV SM criteria	Questionnaire
36	Klein et al	2019	SM	42	2.4–13.8	Comparative (parent/teacher)and correlational one-sample study	ADIS, SMQ	Questionnaire and interview
37	Koskela et al	2020	SM/community controls	860/3250	3–15	Community cohort study	ICD-10 criteria	Observation
38	Milic et al	2020	SM/SAD/Control	25/17/15	3–7	Case–control	ADIS, SMQ, SMQ-C, SSQ, Behavioral observation tasks	Questionnaire, interview and observation
39	Muchnik et al	2013	SM/community controls	31/31	5–16	Case–control observational study	DSM-IV criteria and video/audio at home (no formal instrument)	Observation
40	Muris et al	2017	General population	187	8–12	Cross-sectional	YAM-5, SMQ	Questionnaire
41	Muris et al	2021	General population	172	3–6	Cross-sectional	SMQ	Questionnaire
42	Nowakowski et al	2011	SM/anxiety/typical development comparison groups	19/18/26	5–8	Case–control observational study	SpSQ-P, SpSQ-T	Questionnaire
43	Poole et al	2021	SM/SAD/Control	158	4–16	Multi-method	DSM classification, SM-BCFPI, SpSQ	Questionnaire, interview and observation
44	Schwenck et al	2019	SM-symptoms/SAD-symptoms/communitiy controls	52/28/41	mean age: 13.30 years, (SD = 3.26)	Quasi-experimental, online-based study	FSSM	Questionnaire
45	Schwenck et al	2021	SM	91	3–17	Qualitative, online-based study	FSSM	Questionnaire
46	Sharkey & McNicholas	2012	SM/community controls	20/10	4–12	Cross-sectional prevalence study	SMQ	Questionnaire
47	Starke	2018	SM/community controls	18/12	3–5	Case–control	Dortmund Mutism Screening, Parental questionnaire on mute behavior	Questionnaire
48	Stein et al	2011	SM/anxious/non-anxious group	106/1948	3–11	Clinical cohort study	SMQ, ADIS	Questionnaire and interview
49	Vogel et al	2019	SM/social phobia/typical development	65 /18 /51	mean age: 13.25 years (SD = 3.2)	online survey in 3 groups of children (SM, SAD, TD)	FSSM	Questionnaire
50	Wichstrøm et al	2012	General	2475	4	Prevalence	PAPA	Interview
51	Young et al	2012	SM/social phobia/community controls	10/25	5–12	Case–control study	ADIS	Interview
Psychometric studies								
52	Gensthaler et al	2018	SM/anxious/community controls	95/239	3–18	Psychometric	FSSM, ESCM, Kinder-DIPS	Questionnaire and interview
53	Martinez et al	2015	SM/anxious	19/10	6–11	Case–control, psychometric	ADIS, SMQ, TTI-SM	Questionnaire and interview
54	Oerbeck et al	2020	SM/community control	32/32	3–9	Psychometric	SMQ, SSQ	Questionnaire
55	Olivares-Olivares et al	2021	SM	110	3–10	Psychometric	SMQ, ADIS	Questionnaire and Interview
56	Unal et al	2019	SM/other psychiatric disorders	10/140	6–17	Psychometric	SMQ, K-SADS-PL-DSM-5 SM supplement	Questionnaire and interview

*SMQ*  Selective Mutism Questionnaire, *DBR* Direct Behavior Rating, *ADIS*  Anxiety Disorders Interview Schedule, *SSQ*  School speech questionnaire, *IEBE*  Independent Evaluator Behavioral Evaluation, *SNAP* Strong Narrative Assessment Procedure, *CGI*  Clinical Global Impression, *DRCB*  Daily Rating of Child Behavior (Parent/Teacher), *SM-BOT*  SM-Behavioral Observation Task, *SM-SCCS * SM-Social Communication Comfort Scale, *GAS*  Goal Attainment Scaling, *ESCM*  Evaluation of the Socially interactive Communication in Mutism, *PAPA*  Preschool Age Psychiatric Assessment, *SpSQ* = Speech Situations Questionnaire, *SM-BCFPI*  Selective Mutism—Brief Child and Family Phone Interview, *FSSM*  Frankfurter Scale of Selective Mutism, *TTI-SM*  Teacher Telephone Interview-SM, K-SADS-PL  Kiddie Schedule for Affective Disorders and Schizophrenia–Present and Lifetime Version

^*^ (part of) the same sample was used for multiple studies and publications

In total, these 56 articles describe the use of 29 different instruments (see Table 4).

**Table 4:** Instruments used for the assessment of SM

Instrument	Authors	Respondent	Times used (Study IDs refer to Table 3)	Total number of items	Subscales	Measures severity (scale-level) or classification (cut-off)	Reliability: Internal consistency (Cronbach's Alpha)
Questionnaires							
SMQ	Bergman et al. [17]	Parents*	32; Study ID: 1–5, 7–10, 12–15, 17–21, 24, 25, 29, 30, 33, 36, 38, 40, 41, 46, 48, 53–56	17 items SM total scale; 6 items interference scale	School; home; community	Severity	Psychometric studies: TotSc α = 0.97, SubSc α = 0.97, 0.88, 0.96 [22] TotSc α = 0.78, SubSc α = 0.91, 0.65. 73 [23] TotSc α = 0.96 [29] TotSc α = 0.90 [30]
SSQ	Bergman et al. [5]	Teacher	11; Study ID: 3, 4, 7, 9, 17–20, 25, 38, 54	11	School	Severity	α = .76 [24], α = 0.64 [27], α = 0.84 [28] α = 0.97 [29]
FSSM	Gensthaler et al. [37]	Parent	4; Study ID: 44, 45, 49, 52	Diagnostic scale (10 items); severity scale (41 items in the 3–7 version; 42 items in the 6–11 / 12–18 versions)	Diagnostic Scale, 3 Severity subcales at kindergarten/school, in public and at home	Classification and severity	α = 0.90–0.98
pSQ-P and SpSQ-T	Cunningham et al. [39]	Parents	4; Study ID: 27, 31, 42, 43	SpSQ-P: 15; SpSQ-T: 7	Home, school, community	Severity	α = 0.82 [45], α = 0.92 [46], α = 0.92 [47]
Verbal and Nonverbal Social Interactions Skills	Cunningham et al. [39]	Parent, teacher, child	1; Study ID: 27	Parent: 38; Teacher: 28	Verbal social skills, nonverbal social skills, nonverbal cooperation	-	Parent scales: α =0 .58–0.78, α =0 .71–0.83, and α =0 .78–0.85 for the subscales; Teacher scales: α =0 .80–0.90, α = .86–0.94, and α = 0.88–0.92 for the subscales
Parental questionnaire on mute behavior	Starke [43]	Parents	1; Study ID: 47	18	Family, neighborhood, public	Severity	α =0 .89–0.95
ESCM	Hartmann [38]	-	2; Study ID: 22, 52	23	-	Severity	**–**
Severity of Behavior Form	Mitchell & Kratochwill [45]	Parent, teacher	1; Study ID: 16	–	–	Severity	**–**
Dortmund Mutism Screening	Starke [43]	Teacher	1; Study ID: 47	17	mute behavior + expression of needs, group situations	Severity	Subscale 1: α = 0.92–0.95, Subscale 2: α = 0.89–0.94
Therapist-created questionnaire for DSM-IV SM criteria	Klein et al. [47]	Parents	1; Study ID: 35	–	–	Classification	-
ICL adaptation rating speaking behavior	Oerbeck et al.[23]	Child	1; Study ID: 20	–	–	Severity	-
YAM-5	Muris et al. [49]	Child	1: Study ID: 40	–	n/a	Severity	α =0 .41 (SM segment)
SMQ-C	Milic et al. [50]	Child	1; Study ID: 38	20	School, Home, Public	Severity	Subsc α = 0.67–0.82
Interviews with SM section							
ADIS	Silverman & Albano [51]	Parents	23; Study ID: 3, 5–7, 9, 11, 14, 15, 17–20, 23–25, 29, 34, 36, 38, 48, 51, 53, 55	–	n/a	Classification and severity	–
K-SADS-PL-DSM-5	Kaufman et al. [56]	Parents	1; Study ID: 56	–	n/a	Classification and severity	–
PAPA	Egger et al. [59]	Parents	2; Study ID 26, 50	1	n/a	Classification and severity	–
TTI-SM	Tannock, Fung, & Manassis [62]	Teacher	1; Study ID: 53	54	SM, Verbal/Nonverbal communication with teachers/peers, school and classroom social participation behaviors, externalizing behaviors	Severity	α = 0.96 (SM Subscale)
Kinder-DIPS	Schneider et al. [64]	Parents	3; Study ID: 28, 32, 52	–	n/a	Classification	_
SM-BCFPI	Cunningham et al. [66]	Parents	2; Study ID: 31, 43	–	n/a	–	–
Observation Scales							
SM-BOT	Kurtz [69]	Clinician	1; Study ID: 7	n/a	–	Unspecified	–
SM-SCCS	Shipon-Blum [71]	Clinician	1; Study ID: 14	n/a	4 levels of communication	Severity	–
GAS	Kiresuk, Smith, & Cardillo [72]	Clinician	1; Study ID: 16	n/a	n/a	Severity	–
DRCB-P and DRSB-T	Kearney [73]	Parent, teacher	1; Study ID: 4	–	–	Severity	–
DBR	Barterian [74]	Parents	1; Study ID: 2	3	Social engagement, spontaneous speech, responsive speech	Severity	–
IEBE	Bergman et al. [19]	Clinician	1; Study ID: 3	–	–	–	–
SNAP	Strong [75]	Clinician	2; Study ID 3, 7	–	–	–	–
RBOCSM- adapted version	Mitchell & Kratochwill [45]	Parent, teacher	1; Study ID: 16	–	–	–	–
Structured parent–child interaction play	Edison [67]	Clinician	1; Study ID: 31	–	–	–	–
Shaping hierarchy of speaking behavior observational measure; frequency of target behavior	Bunnell [78, 79]	Clinician	1; Study ID 5, 6	–	–	–	–

*SMQ * Selective Mutism Questionnaire, *SSQ*  School Speech Questionnaire, *FSSM*  Frankfurter Scale of Selective Mutism, *SpSQ*  Speech Situations Questionnaire, *ESCM*  Evaluation of the Socially interactive Communication in Mutism, *ADIS*  Anxiety Disorders Interview Schedule, *K-SADS-PL* Kiddie Schedule for Affective Disorders and Schizophrenia–Present and Lifetime Version, *PAPA*  Preschool Age Psychiatric Assessment, *TTI-SM*  Teacher Telephone Interview-SM, *SM-BCFPI* SM Brief Child and Family Phone Interview, *SM-BOT*  SM-Behavioral Observation Task, *SM-SCCS*  SM-Social Communication Comfort Scale, *GAS* Goal Attainment Scaling, *DRCB*  Daily Rating of Child Behavior (Parent/Teacher), *DBR * Direct Behavior Rating, *IEBE* Independent Evaluator Behavioral Evaluation, *SNAP* Strong Narrative Assessment Procedure, *RBOCSM*  Revised Behavioral Observation Code for Selective Mutism

n/a: not applicable

-: unspecified

^*^: the SMQ was used as a self-report in Study ID 40

## Selective mutism assessment measures

### Questionnaires

The *Selective Mutism Questionnaire (SMQ *[17]*)* was used most often (in 32 articles, see Table 3, Study ID: 1–5, 7–10, 12–15, 17–21, 24, 25, 29, 30, 33, 36, 38, 40, 41, 46, 48, 53–56).

The SMQ is a parent rating scale, measuring the severity of SM rated speaking behavior in different contexts (17 items) and impediment associated with nonspeaking behavior (six additional items). Items are scored on a 4-point Likert scale ranging from 0 (never speaking) to 3 (always speaking). The SMQ was developed and validated in the United States, in 3- to 11-year-old children with and without SM. Exploratory Factor Analyses revealed a three-factor structure; speaking in the context of: school (6 items), home/family (6 items), and community (5 items) [17, 18]. The three-factor structure was confirmed in an independent study but only after removing 4 items [18]. Internal consistency was good–excellent in the original sample (*α* = 0.97 total scale, subscales respectively *α* = 0.97, 0.88, 0.96) [17] and moderate–excellent in above-mentioned independent clinical and community sample, based on the 13-item version (*α* = 0.78 total scale, subscales *α* = 0.91, 0.65, 0.73) [18]. The internal consistency of the total scale ranged from *α* = 0.70 to 0.91 in subsequent samples in the US [19–21]. The SMQ was translated into different languages. Studies in the Norwegian population reported internal consistencies ranging from *α* = 0.77 to 0.93 on the Total scale and *α* = 0.68 to 0.90 on the subscales [22, 23]. A recent study by Oerbeck et al. reported internal consistency of *α* = 0.96 on the total scale in a clinical sample and a typically developing control group [24]. The internal consistency of the total SMQ scale in a Spanish clinical sample was found to be *α* = 0.90 [25]. Additionally, the three-factor structure as found by Bergman et al. [17] was replicated. Furthermore, the SMQ has been translated and used in Italy [26, 27], Israel [28, 29] (reported Cronbach’s *α* = 0.77), and Turkey ([30] Cronbach’s *α* = 0.87).

Convergent validity of the SMQ was indicated by significant correlations with two (social) anxiety scales (SASC-R and MASC-P) for children and with the ADIS severity score (*r* = − 0.67) [17]. Letamendi et al. found moderate correlations between the 13-item SMQ total severity scale and the interference scale and the clinician rated ADIS severity score (respectively, *r* = 0.42 and *r* = 0.48) [18]. Discriminant validity was supported by a weak non-significant correlation with the Harm Avoidance, Separation Anxiety, and Physical Symptoms subscales of the MASC [17]. However, in a previous study, the total SMQ scale did not differentiate 14 SM children from 9 children with social anxiety disorder [31]. Finally, the SMQ was found to be sensitive to treatment change in small samples of children with SM [17, 32–34]. Incremental validity of the 13-item SMQ was supported by a significant additional explained variance in SM diagnosis over Anxious/Depressed Syndrome Scale of the CBCL [18].

The *School Speech questionnaire (SSQ *[5]*)* is a modified version of the SMQ measuring speech at school as rated by the teacher. It was used in 11 publications (see Table 3, Study ID: 3, 4, 7, 9, 17–20, 25, 38, 54). The original version comprised 11 items, but 2 items related to nonverbal communication with low item-total correlations were dropped. Internal consistency of the 9-item version was *α* = 0.96 [5]. In subsequent studies, 6–10 item versions have been used, but authors provide no explanation for the decision to use certain items. The first 6 items are identical to the parent-rated SMQ school items and form the Total severity scale. The items are scored on the same 0–3-point scale as the SMQ. The additional items address nonverbal behavior and interference with academic and social functioning and are often not used in analyses [21–23, 33–36]. Internal consistency ranged between *α* = 0.76 and 0.81 in Canada and the US, and between α = 0.64 and 0.84 in Norway. The SSQ was found to be sensitive to treatment change [19, 21, 22, 34].

The *Frankfurter Scale of SM (FSSM *[37]*)* was validated in a German-speaking clinical sample (SM *n* = 95, social phobia *n* = 74, internalizing problems *n* = 46), and a community sample (*n* = 119) for use in children ages 3–18 (see Table 3, Study ID: 52). The FSSM was used in subsequent publications (see Table 3, Study ID: 44, 45, 49). The FSSM has three age versions: 3–7, 6–11 and 12–18 years. The questionnaire is freely available online in English, German, Norwegian and Finnish. It comprises a diagnostic scale (10 items, yes or no) and a severity scale (41 items in the 3–7 version; 42 items in the 6–11 and 12–18 version). The severity subscale measures speaking in three contexts: school, public and home (5-point Likert scale; 0 = speaks without problems, to 4 = speaks not at all). Internal consistency of the diagnostic scale was α = 0.90 (FSSM 3–7, FSSM 12–18) and 0.92 (FSSM 6–11). Internal consistency of the total severity scale was excellent (*α* = 0.98) in the total (clinical and community) sample and good (*α* = 0.88) in the SM group.

Exploratory factor analysis of the severity scale showed a one-factor solution. ROC curve analyses of the diagnostic scale revealed an optimal cutoff score to differentiate the SM group from the group with social phobia, other internalizing disorders and the non-clinical group. Areas under the curve were between 0.94 and 0.99 for the three age versions, indicating satisfactory to excellent discriminant validity. Convergent validity based on correlations with Evaluation of the Socially interactive Communication in Mutism scale developed by Hartmann [38] showed significant correlations with the total score on the severity scale (*r* = 0.48–0.72), indicating good convergent validity.

The *Speech situations questionnaire (SpSQ-P and SpSQ-T)* was used in 4 publications (see Table 3, Study ID: 27, 31, 42, 43). The questionnaire for parents comprises 15 items and measures the extent to which the child speaks to different people in different settings including home, school, and community. It is scored on a 3-point scale (0 = never talks, 1 = whispers, 2 = talks normally). The teacher version is scored on the same scale and comprises 7 items asking about speech in different situations and people at school. Internal consistency of the SpSQ-P was found to be good in the original sample of 58 children with SM and 52 community control children (Cronbach’s *α* = 0.82) ([39], and excellent in subsequent studies *α* = 0.92 [40–42]. Internal consistency of the SpSQ-T was excellent (Cronbach’s *α* = 0.95–0.96 [40, 42]. No further psychometrics are reported on these instruments.

The *Verbal and Nonverbal Social Interactions Skills* [39] was used in one study (see Table 3, Study ID: 27) [40]. The scale was derived from the SSRS-Parent and SSRS-Teacher, first described by Cunningham and colleagues [39] and comprises three subscales: verbal social skills, nonverbal social skills, and nonverbal cooperation. The internal consistency in the clinical and community samples of Carbone [37] and Cunningham [38], respectively, ranged from Cronbach’s *a* = 0.58 to 0.78, *a* = 0.71 to 0.83, and *a* = 0.78 to 0.85 for the three scales in parents. The same three subscales were computed in teachers and internal consistency ranged between Cronbach’s *a* = 0.80–0.90, *a* = 0.86–0.94, and *a* = 0.88–0.92 for these three scales. No further psychometrics have been reported.

The *Parental questionnaire on mute behavior * [43] was used in one study (see Table 3, Study ID: 47[43]). The questionnaire comprises 18 items. Speaking behavior was rated on a 5-point Likert scale. The items covered social situations in the family context, neighborhood, and in public. In the study, the instrument was used at four time points within one year and the internal consistency for the total scale was good to excellent (ranging from: *α* = 0.89 to 0.95 over the four time points). No further psychometrics have been reported.

The *Evaluation of the Socially interactive Communication in Mutism (ESCM *[38]*) *is a 23-item questionnaire measuring verbal and nonverbal communication in different situations, rated on a 3-point Likert-type scale (0 = uninhibited communication, 1 = communication moderately stressful or on request only, 2 = stressful/selectively mute). It has been used in two studies (see Table 3, Study ID: 22, 52) to investigate symptom severity in the study of Gensthaler et al. [37] and to investigate improvement over time and treatment outcome in the case study of Plener et al. [44]. The ESCM is online available in German and English. No further psychometrics have been reported.

*Other questionnaires *were used in six studies. Each questionnaire was used only once and the instruments have been minimally described. Mitchell and Kratochwill [45] used a Severity of Behavior Form derived from items from the Parent/School Screening Questionnaires [46]. The form was used on a weekly basis to investigate change over time, but the items were not described in the article. The Dortmund Mutism Screening [43] is a 17-item preschool teacher-reported questionnaire used in one study by Starke [43] and is available in German only. In the study of Klein et al., a therapist-created questionnaire for DSM-IV SM criteria was used, but not further described (see Table 3, Study ID: 35)[47].

A few studies report the use of self-report measures. Oerbeck et al. [23] described using an adaptation of the Inventory of Life Quality in Children and Adolescents (ILC) [48] where children could rate their difficulties with speaking at school/outside/home on a Likert scale (1 = very easy, 3 = mixed, 5 = difficult). No further psychometrics have been reported. In two studies, the SMQ was used as a self-report measure. Muris et al. [49] described that children rated the SMQ items on a four-point scale (0 = totally disagree, 1 = somewhat disagree, 2, somewhat agree, 3 = totally agree), reporting Cronbach’s *α* = 0.82 in a non-clinical sample. The study of Muris et al. also used the Youth Anxiety Measure for DSM-5 (YAM-5). The YAM-5 is a questionnaire for children aged 8 to 18 years and their parents. The self-report version is answered on a Likert scale (0 = never, 1 = sometimes, 2 = often, and 3 = always). Muris et al. reported Cronbach’s *α* = 0.41 for the SM segment and a correlation of *r* = -0.63 for the SM segment and the SMQ [49]. In the study of Milic et al. [50], the SMQ-Child (SMQ-C) was introduced as a 20-item measure of frequency of speech, using a two stage pictorial response scale. Internal consistency is reported for the subscales (*α* = school 0.82, home/family 0.67, public/social 0.80), referring to an unpublished doctoral thesis. No further psychometrics have been reported.

### Clinical interviews

The *Anxiety Disorders Interview Schedule—SM section (ADIS *[51]*)* was used in 23 publications (see Table 3, Study ID: 3, 5–7, 9, 11, 14, 15, 17–20, 23–25, 29, 34, 36, 38, 48, 51, 53, 55). The ADIS is a clinician-administered, semi-structured interview to assess the presence of a broad range of anxiety, mood and behavioral disorders, consisting of a parental and a child interview version. The SM subsection is part of the parental interview and encompasses 8 items, questioning the speaking behavior of the child and the school functioning (i.e., dysfunction related to difficulties with speaking at school). Parents rate on a 9-point scale (i.e., 0–8) to what extent the symptoms interfere with the daily life of the child. The interviewer rates the level of impairment on the same 9-point scale (Clinician Severity Rating, CSR), with a CSR of 4 or more, indicating a clinically significant problem. The interview has good reliability and validity ([29] [52]). The ADIS (adapted to the DSM-IV criteria) is frequently used in studies on SM to assess comorbid (anxiety) disorders. However, specific information about the psychometric properties of the ADIS to differentiate SM from other anxiety disorders has not been reported. The SM section is mostly used to assess the classification SM for inclusion in the study. In seven studies, the CSR was used as a measure of severity or outcome measure [19, 21, 27, 29, 34, 53–55]. In these studies, the ADIS was sensitive to treatment change. Good inter-rater reliability between the ADIS and the clinical global impression scales was found [29].

The *Kiddie Schedule for Affective Disorders and Schizophrenia–Present and Lifetime Version (K-SADS-PL*[56]*)* has been used in studies into SM to assess comorbidity. Until recently SM could not be investigated with the K-SADS-PL. The latest version (K-SADS-PL-DSM-5; [57]) has a SM supplement. The reliability and validity of the SM supplement was found to be excellent in a Turkish study that included 10 children with SM (see Table 3, Study ID: 56) [30]. In addition to the studies identified in our search, a recently published Japanese research group (n = 4) found good criterion validity, adequate construct validity and excellent convergent validity was for the SM supplement. Due to the very small sample size, these results should be interpreted with great caution [58].

The *Preschool Age Psychiatric Assessment (PAPA *[59]*) *is a clinical DSM-screening interview used in two studies (see Table 3, Study ID: 26, 50)[60, 61]. SM is screened using a single item rated as 0 = SM is absent, 1 = limited speech/volume or 2 = absence of speech in specific situations. The interview is available online: Preschool Age Psychiatric Assessment 2.0.7 (duke.edu).

The *Teacher Telephone Interview (TTI-SM *[62]*) *is described and evaluated by Martinez et al. (see Table 3, Study ID: 53) [63] to assess SM and anxiety in the school. The authors refer to an unpublished article of Tannock et al. [62] as a source of the interview. The TTI-SM is a standardized phone interview that can be conducted with teachers to include the perspective of the teacher in the assessment of SM. The TTI-SM comprises five subscales: SM (15 items), Verbal/Nonverbal Communication with Teachers (14 items), Verbal and Nonverbal Communication with Peers (10 items), School and Classroom Social Participation Behaviors (8 items), and Externalizing Behaviors (7 items). Martinez et al. [63] only evaluated the SM subscale and the School and Classroom Social Participation Behaviors subscale. The items are shown in their article (p. 90–91). After exclusion of four items from the SM subscale due to low item-subscale correlations, the internal consistency of the 11-item subscale was excellent (Cronbach’s *α* = 0.96). Convergent validity of the TTI-SM subscale was indicated by significant correlations with the SMQ (*r* = 0.72 and *r* = 0.85 for the mother and father reported SMQ). Discriminant validity was supported by low–moderate non-significantly correlation with the total SASC-R and MASC total scores, although there were some correlations on subscale levels. Concurrent validity was supported by significant differences on the TTI-SM subscale between 33 children with SM and 10 children with mixed anxiety disorders. Predictive validity was supported by a significant correlation between the TTI-SM subscale and the ADIS classification [63].

*Other interviews *were briefly described and are summarized here. A German online open access DSM-interview (Kinder-DIPS, [64]) was used in three studies. This interview is not available in other languages (see Table 3, Study ID: 28, 32, 52)[65]. The *SM Brief Child and Family Phone Interview (SM-BCFPI*, [66]) was used by Edison et al. [67] and Poole et al. [68] (see Table 3, Study ID: 31, 43). This is a SM-specific version of the BCFPI. As far as known to the authors, the BCFPI does not encompass a scale for SM. It has not been described how the BCFPI was adjusted to assess SM in their study.

### Observation measures

The *SM-Behavioral Observation Task* (*SM-BOT *[69]*)* was used in one treatment evaluation study (see Table 3, study ID: 7) [34]. To refer to the SM-BOT, the authors of this study cite Carpenter et al. [70], who described the SM-BOT as an instrument to assess parent–child interactions. In this context, Carpenter et al. refer to an unpublished manuscript from Kurtz [69], without further clarification of this instrument. In a publication of Catchpole [34], the SM-BOT is coded on the basis of an interaction task involving parent–child interactions in the presence and absence of a stranger and child-stranger interaction tasks. The child’s behavior is coded as having: (a) no interaction with the stranger, (b) non-verbal interaction only, or (c) non-verbal and verbal interaction. In sum, no further description or psychometric information on the SM-BOT was found.

The *SM-Social Communication Comfort Scale (SM-SCCS *[71]*)*: is used in one study (see Table 3, Study ID: 14) [54]. The SM-SCCS is a clinician-administered observation scale that can be retrieved from the website of the Smart Center [71]. Klein et al. [54] describe the instrument for use in the therapy sessions, for assessing therapist-child interactions and progress. Four stages of communicative behavior are distinguished, each comprising 2 levels (responding and/or initiating): stage 0 no communication, stage 1 non-verbal communication, stage 2 transition into verbal communication (whispering, making sounds) and stage 3 verbal communication. No psychometric properties were mentioned.

The *Goal Attainment Scaling (GAS *[72]*)* was used in one study to monitor progress of an intervention targeting SM (see Table 3, Study ID: 16). The GAS allows for quantifying progress toward a specific individualized goal. Usually GAS-scales comprise 5 points with 0 meaning no change, negative scores indicating deterioration and positive scores meaning improvement. In the study of Mitchell and Kratochwill into SM [45], the GAS-scale used ranged from: − 2 (failure to speak) to + 2 (normal speech in all situations) and was rated by parents and teachers.

The *Parent’s Daily Rating of Child Behavior (DRCB-P) *and *Teacher’s Daily Rating of Student Behavior (DRSB-T *[73]*) *were used by Bork & Bennet [33] describing three children with SM (see Table 3, Study ID: 4). The original goal was to ask parents and teachers to record the frequency in number of words and the volume of speech (on a 0–10 audible scale) on a daily basis. However, it appeared to be too difficult for parents and teachers to report the exact number of words. Instead they were then asked to record new verbal behaviors (e.g., speaking to a new person, spontaneous speech instead of on request). There is no psychometric information available for these rating scales.

A *Direct Behavior Rating measure* was used by Barterian et al. [74] (see Table 3, Study ID: 2) to measure speaking behavior and social anxiety symptomatology in naturalistic situations three times per week. Parents were trained and asked to observe their child during social interaction with an unfamiliar adult. Frequency and ease of social engagement, and spontaneous and responsive speech were rated on a 10-point scale (from 0 = never to 10 = always).

An *Independent Evaluator Behavioral Evaluation (IEBE *[19]*)* of verbal and non-verbal behavior during 10 structured interactional tasks was used by Bergman et al. [19] (see Table 3, Study ID: 3). It can be administered during a 10–15 min playful interaction. No further psychometric information was provided.

The *Strong Narrative Assessment Procedure-Retell (SNAP*[75]*)* is a standardized narrative elicitation task and was adapted to assess narrative abilities in children with SM [76]. It was used in two studies (see Table 3, Study ID: 3 and 7) as a treatment outcome measure. In this task, the teacher was asked to read a storybook to the child and the child was asked to retell the story. The narrative of the child was audio-recorded, so that the length of the story could be analyzed. The SNAP appeared to be sensitive to change in this study. The psychometric properties of the task are unknown [19].

*Other observational measures *were minimally described and are summarized here. In the study of Mitchell and Kratochwill [45], parent and teachers were asked to rate the number of spoken words and independent raters were mobilized twice a week to code the words spoken during classroom one-hour observations. The procedure was adapted from the Revised Behavioral Observation Code for Selective Mutism (RBOCSM; [77]). The adaptations or coding schemes were not described [45]. Edison et al. [67] coded speech from video during parent–child interaction play in a laboratory setting. Bunnell and colleagues used video software to observe and analyze treatment process by means of percentage of time that the child spoke during a task [78] and the latency to respond to communicative prompts [79].

### Other instruments

A small number of studies used severity scales originally designed to measure general functioning, to specifically assess severity or improvement of the core speaking symptom of SM (criterion A). The Global Assessment of Functioning (GAF, [80]) or the Children’s Global Assessment Scale (CGAS, [81]), rated on a 0–100 scale, were adaptively used to assess SM severity [55, 60, 82] and improvement after treatment [21, 44, 70]. Accordingly, the Clinical Global Impression—Severity (CGI-S) and Improvement (CGI-I) Scales [83] were used to investigate specific SM behavior and behavioral change. The scales are rated on 7-point scales. The CGI-S ranges from “Not At All Ill” to “Extremely Ill”, and the CGI-I ranges from “Very Much Improved” to “Very Much Worse”[19, 21, 29, 35, 36, 70, 74]. Finally, the teacher version of the Impairment Rating Scale (IRS; [84]) was used to measure specific impairment at school as a result of SM. The IRS has 8 items and is measured on a 7-point scale (0 = no problem tot 6 = extreme problem)[21].

## Discussion

The aim of this systematic review was to identify and evaluate the assessment instruments that have been used in the past decade for the purpose of screening and assessing severity of SM symptomatology, classification of the disorder and monitoring treatment outcomes on speaking behavior. The number of studies on SM increased over the past years, and studies with larger samples are emerging. However, the majority of samples are still small. To enhance insight in SM and treatment outcomes, comparability of methodology and instruments between groups is utterly important.

Our review revealed that the majority of studies published in the last decade, used one or more standardized or quantified measures. Interestingly, 38% of studies did not use any standardized diagnostic instrument for SM.

To classify SM in clinical practice or to confirm the diagnosis for the purpose of inclusion in research, questionnaires and clinical interviews have been used. The measure used most often in research is the SMQ [17]. This measure is short and comprises a severity scale investigating speaking in three different contexts and a scale to investigate interference of the symptoms on the child’s and family functioning. The reliability of the measure has been assessed in different cultural samples, showing acceptable to excellent internal consistency [19–21, 24–30]. There is support for adequate convergent, discriminant and incremental validity [17, 18]. Because of the few studies to date, there is limited validity. The questionnaire had been translated into different languages and publications represented in the present review came from the US, Norway, Italy, Israel, Turkey and Spain. Although developed for 3–11-year-old children, the SMQ has been used in older children as well. However, some of the items may not be appropriate for the very young children or for older adolescents. In future research, it is important to further investigate the correlation of SM severity and age. In most studies, the majority of children are young and larger SMQ datasets about adolescents are lacking. The SMQ does not provide cut-off scores and is therefore not easily applicable to classify groups, although some studies use the mean and standard deviation of the SM group in the original sample. Most studies used a clinical interview (ADIS) in addition to the SMQ to confirm the clinical diagnosis [19]. The SMQ can be used to differentiate between children with and without SM [24]. The use of cut-off scores, and the use of the SMQ in typically developing children (TDs) can be investigated in larger samples. Research is emerging but still quite limited.

An interesting questionnaire for teachers is the SSQ, adapted from the SMQ [5]. The number of items varies somewhat between studies, without further explanation in the articles. Differing item numbers of the SSQ can hamper the comparability of results. Internal consistency varied from questionable to acceptable [19, 21, 22, 36]. The low number of items is both an advantage for teachers who often struggle with time and a disadvantage because only a few situations in school are investigated. The SMQ and SSQ were used in most treatment studies and were found sensitive to treatment changes [19, 22, 34]. Detailed psychometric information is limited regarding non-English speaking samples, the teacher rated SSQ, and also regarding cut-off scores and the use of the SSQ in typically developing children (TDs).

More recently, the FSSM has been developed [37]. The FSSM includes both a diagnostic scale that can be used to classify groups and severity scales that can be used to measure symptom changes. The FSSM has three age versions (3–7, 6–11 and 12–18 years), with items that have been adapted to the developmental stages of the children. The questionnaire is longer than the SMQ (51 items in the preschool version and 52 items in the other versions). The FSSM is a parent-rated measure and parents are encouraged to ask the teacher for help if they have difficulties answering questions about the speaking behavior of their child in school or kindergarten. This may be a disadvantage in treatment studies when an independent teacher rating is wanted. The psychometric properties of the measure are promising with high internal consistency and good convergent and discriminant validity. With the diagnostic scale a SM group was successfully differentiated from children with social phobia and other internalizing disorders. The questionnaire is freely available online in different languages. The other questionnaires evaluated in this review have been less often used, are less well described and/or the psychometric properties have been insufficiently investigated and reported. The advantage of the FSSM is that different age groups are divided. The advantage of the SMQ is that it is used widely, but studied in a young age group.

The clinical interview most often used to investigate the presence and severity of SM, as well as comorbid disorders, is the ADIS [51]. The ADIS has most often been used for inclusion in studies. The severity rating has also been used in seven studies as an outcome measure and was found to be sensitive to change [21, 29, 53]. An alternative for the ADIS is the K-SADS-PL-DSM-5 [57]. Previous versions of this instrument did not have a SM section but the newest version has a SM supplement. The reliability and validity of this SM supplement was found promising in Turkey and Japan [30, 58], but needs to be investigated further in other countries.

For very young children, who fall below the age ranges of the ADIS (4–18 y) and the K-SADS-PL-DSM-5 (6–18), the PAPA; [59] can be used, which is available online. Using PAPA, SM is screened using a single item. Finally, the TTI-SM [62] is of interest to get clinical information from the teacher using a standardized phone interview. Promising psychometric properties have been found in one study for the 11-item SM subscale with high internal consistency and good convergent, predictive and concurrent validity [63]. These psychometrics have to be confirmed in larger new studies in other countries.

Several observational instruments for speaking behavior were developed, but most measures are used in one study only and psychometric information is lacking. Multiple measures comprise the observation and coding of speaking behavior in controlled laboratory settings (SM-BOT; [69], IEBE; [19], SNAP; [75], parent–child interactive play; [67], communicative behavior during interaction tasks; [78, 79]). With the exception of the SNAP story-tell task for children aged 6–13 years, these tasks were too briefly described so that replication of findings or the use in other studies is not possible. Other observation tasks have been designed to rate speaking behavior in daily situations at home or at school (DRSB; [73], DBR; [74], Adapted RBOCSM; [45]). These scales allow for repeated measurements over time for single case experimental designs. The Direct Behavior Rating (DBR) measure described by Barterian et al. [74] is probably the most feasible procedure in naturalistic situations. Hierarchy scales such as the SM-SCCS [71] can also be used to observe and rate behavior for naturalistic situations and to investigate treatment outcome. Of interest is the Goal Attainment Scaling (GAS; [72]). This measure allows for setting individual goals and measuring improvement over time. Finally, other widely used general measures to assess global functioning, impairment and improvement (GAF; [80], CGAS; [81], CGI-S/I; [83]) have been used in several studies as severity and treatment outcome measures [19, 21, 29, 35, 36, 44, 55, 60, 70, 74, 82]. When choosing an observational measure for a study, the observer as well as the setting can be taken into account. However, all observational instruments need more investigation and validation to be valuable measures in clinical practice and research.

Unfortunately, self-report measures for SM are still scarce. Although children with SM often are very young, making parent and teacher ratings necessary, it is known from research that SM can take a chronic course and also affects adolescents and to a lesser extent even adults [9, 10, 85]. The SMQ appears to be a promising measure for self-report as has been shown by Muris et al. in a non-clinical population (8–12 years old) [49] and Milic et al. with an adapted pictorial version in young children (3–7 years old) [50]. We recommend further studying the use of validated SM-specific self-report measures for use in clinical practice and research. In children with SM and/or social anxiety, it was found that parents and clinicians often report higher anxiety levels than the children themselves [55, 86]. Difficulties to speak at school may be more reliably reported by adolescents themselves than by parents or secondary school teachers.

We consider the results of this review as a plea for multi-informant diagnostics using questionnaires, interviews and observation. Although the importance of a multi-informant approach in the assessment of SM has been underlined [55, 86, 87], in many of the reviewed studies only one informant is asked to complete a standardized quantitative measure. To obtain clearer insight in SM and its underlying mechanisms, a multi-informant approach is recommended. We recommend the use of parallel questionnaire or observational versions (parent, teacher, clinician and attuned age-adequate self-reports). As to the content of SM assessment instruments, we recommend to extend and include the range of social settings in which children find themselves (school, after school activities, swimming lessons, play dates, playgrounds, family occasions, shops or restaurants etc.).

This review showed an important shortcoming that over a third of studies (38%) did not report any objective measure to classify SM. This limits the replicability of these studies and psychometrically sound foundation of instruments to be used in clinical practice. Few studies described psychometric properties or validation of the reported instruments in detail.

Another problem was that the instruments used were not always validated for the same population as they are used in (as to age, general vs. clinical population). The majority of measures has not been evaluated in different samples. This may hamper the conclusions since it is not warranted to generalize the applicability of an instrument to another population than it is aimed for, without studying the psychometrics and validity in that population. As there is still a marked lack of evaluated measures, we recommend that a selection of instruments, such as one or two promising questionnaires, parent and teacher interviews and direct observational measures, will be used in combination more often. A smaller set of instruments that are used across studies will increase comparability, facilitate replication and move the field of SM research forward. Furthermore, it is important to note that the instruments used most frequently, were still based on the DSM-IV. However, since core criteria for SM were not changed in the DSM-5 version, we consider our findings as still clinically relevant.

Finally, very little is known about cultural differences in SM characteristics and cross-cultural appropriateness of the instruments used, especially regarding discrepancies between western and different non-western cultures. This is not surprising considering the low prevalence of SM. SM specifically and taciturnity in general may be unrecognized, underreported or perceived from a different perspective in different areas of the world. In this context, we refer to our previous reaction to a Turkish article focusing on speech delay where the diagnosis of selective mutism appeared to be overlooked [88, 89]. It is encouraging to see that studies into SM are emerging and that some of the measures are translated into different languages. Even more encouraging is the fact that several newly developed and validated measures are open access and can be freely derived from the internet. That enlarges the possibilities for researchers around the world to collect and share data.

## Conclusion

This systematic review shows that the SMQ and the teacher-version SSQ [5, 17] are the questionnaires used most often for SM severity ratings. The FSSM is a promising new questionnaire providing a subscale for classification and severity subscales for SM [37]. As to clinical interviews investigating DSM criteria for SM, the ADIS is used most often and also provides a severity rating. There is a need for a well-validated standardized observational measure that can be used in daily situations by parents and teachers. The vast majority of instruments used in different studies were used only once, hampering comparability of findings over studies. In a large part of the articles, no instruments were described at all. We recommend the use of multi-informant measures to investigate SM behavioral characteristic and to classify the disorder, both in clinical practice and in research studies. Several measures with strong or promising psychometrical qualities are available. The use of these measures in different settings allows comparability among studies and will help to understand cross-cultural differences. In addition, we stress the importance of investigating the usability and psychometric properties of self-report measures and observational measures for children and adolescents with SM. For future research, we recommend measures with a broad age range to enable long-term follow-up studies.

## Data Availability

Not applicable.
